# Alarming Dental Hemorrhage

**Published:** 1869-06

**Authors:** A. J. Eidson

**Affiliations:** Colchester, Ill.


ARTICLE XXIII.

### ALARMING DENTAL HEMORRHAGE.

By A. J. EIDSON, M.D., Colchester, Ill.

On the morning of July 5th, 1868, at two o'clock A.M., I was called to see Colombus H., aged 18, of sanguine temperament, good physical development, and by occupation a farmer. He had suffered from odontalgia for a week, and on the previous day had had the middle and posterior molars of the right side of the *os maxillare inferius* extracted by a dentist; one at 10 A.M., the other at 3 P.M. There was considerable hemorrhage from the first, which became augmented when the second tooth was drawn. The young man returned home at 6 P.M.; the bleeding continued without abatement. Domestic remedies were used, *viz.:* salt, alum, and cold water, without effect; and professional aid was deemed indispensable.

I found the family in a state of anxiety and alarm. The patient was lying on a pallet on the floor. The pillow was saturated with blood. A large cloth with which he wiped his mouth was deeply dyed with the *liquor sanguinis.* The floor was spattered for several feet around the bed with mouthfuls of coagula. In a vessel was contained more than a pint of the life-current. From loss of blood and rest, the patient was much inclined to sleep, which, however, was only obtained in snatches, on account of the accumulation of coagula and threatened strangulation.

The appearance of the patient was striking. There was jactitation and subsultus; the face pale and haggard; pulse 90, weak and fluttering. Every 15 or 20 minutes he had partial fainting, with nausea, at which times the bleeding abated, only to increase when the system rallied.

I ordered a compress of carded cotton, soaked in a saturated solution of acid tanicum, laid on the gums, to be firmly retained by forcibly closing the jaws. This checked the flow for a short time. Firm pressure on the carotid artery moderated the hemorrhage. Not having a more powerful styptic at hand, I broke up the coagula in the bleeding sockets, and with a small pointed syringe injected the *tinct. ferri chlo.*, as near the seat of hemorrhage as possible. I then saturated a fresh compress of cotton with the tincture, and applied as the first, enjoined the patient to make firm pressure with the lower jaw. The bleeding was at once arrested, but returned in about an hour. A fresh compress, prepared as before, was applied, and the bleeding ceased for three hours, when it partially returned. The cotton and superficial coagula were removed; another application of the remedy as before, and the hemorrhage was permanently arrested. Rest and quiet were enjoined, and a nutritious diet ordered,.with the following:—

℞. Tinct. Ferri Chlo., S. M. x. Three times a day.

This to be followed in four or five days with—

℞. Elixir Calisa. Ferrat., S. ʒiv. Three times a day.

Patient's recovery somewhat tedious, but satisfactory and complete.

I ascertained that both parents of the young man were of the hemorrhagic diathesis, and that every member of the family was prone to bleed much and persistently from slight abrasions. None of them, except the patient, had ever had teeth extracted at adult age.

